# Development and Feasibility of an Online Brief Emotion Regulation Training (BERT) Program for Emerging Adults

**DOI:** 10.3389/fpubh.2022.858370

**Published:** 2022-06-10

**Authors:** Alyssa Jo Gatto, Truitt J. Elliott, Jonathan S. Briganti, Michael J. Stamper, Nathaniel D. Porter, Anne M. Brown, Samantha M. Harden, Lee D. Cooper, Julie C. Dunsmore

**Affiliations:** ^1^Department of Psychology, Virginia Tech, Blacksburg, VA, United States; ^2^University Libraries, Virginia Tech, Blacksburg, VA, United States; ^3^Department of Sociology, Virginia Tech, Blacksburg, VA, United States; ^4^Department of Human Nutrition, Foods, and Exercise, Virginia Tech, Blacksburg, VA, United States; ^5^Department of Psychological, Health and Learning Sciences, University of Houston, Houston, TX, United States

**Keywords:** emotion regulation, emerging adults, technology, intervention, implementation science

## Abstract

Mental wellness is a critical component of healthy development in emerging adulthood and serves to protect against stress and promote resilience against psychopathology. Emotion regulation is a key mechanism for effective prevention because of its role in socio-emotional competence and its transdiagnostic significance for psychopathology. In this feasibility study, a brief, time and cost-effective emotion regulation training program for emerging adults (BERT) was developed and tested using the RE-AIM framework. Importantly, building interventions within the context of an implementation framework, such as the RE-AIM framework, enhances the chances that an intervention will be able to scale out and scale up. First, the brainwriting premortem method was utilized to refine program content, conducting focus groups a priori to identify potential program failures prior to program implementation. Undergraduate students (*n* = 12) attended four focus groups presenting initial program content. Four clinicians were also interviewed to determine program barriers. Qualitative analyses aggregated participant feedback to identify compliments, changes, and concerns about BERT and critical feedback was immediately implemented prior to initial testing. BERT was rooted in cognitive-behavioral practices and informed by the Gross model of emotion regulation. The 5-week program was then examined in a college sample (*N* = 42) to evaluate implementation (low attrition, high content engagement, favorable attitudes, low incidence of technical errors, costs), reach (enrollment and completion demographics comparable to the population in which recruitment took place), and efficacy (positive change in emotion regulation pre- to post-program). Of the recruited participants, 36 remained in the study where 27 completed at least 80% of program content. Repeated-measures ANOVAs exhibited significant improvements in emotion regulation, psychological distress, and negative affectivity, suggesting promising initial efficacy. Initial data provide support for feasibility and a future randomized control trial. BERT has potential significance for promoting healthy development as its brief electronic format reduced barriers and the program development process incorporated stakeholder feedback at multiple levels to inform better implementation and dissemination.

## Introduction

Mental wellness is a critical piece of healthy development in emerging adulthood, a time when youth transition from adolescence to adulthood (1, 2). For optimal functioning, emerging adults must utilize appropriate and effective regulatory strategies (3). Healthy emotional functioning is particularly relevant to this population as they take on new roles, solidify identities, and face more responsibility (4–6). This developmental period brings unique stressors and increased independence, making it difficult to translate skills from late adolescence into early adulthood (7, 8). This “perfect storm” is evident in the transition to college, when students often leave home for the first time facing new expectations for their socioemotional and executive functioning furthering inconsistent mental wellbeing (9–11).

Most college students are emerging adults [18-26 years of age] and are likely to experience violence, accidents, and substance use as well as psychological distress based on the collegiate schedule [i.e., multiple exams, course load, financial obligations; (12, 13)]. Without skills to cope with these stressors, emerging adults experience elevated risk for chronic illness, suicide, and addiction (14–16). Emotion regulation (ER), which encompasses responses to emotions and the ability to control emotion processes, is a foundational skill to target as it is critical for healthy development (17). ER deficits are associated with multiple disorders including depression, anxiety, substance-use, personality, eating, and somatoform disorders (18). Building ER skills through brief intervention has the advantage of targeting a common mechanism and resiliency factor underpinning multiple disorders, thereby broadening the range of impact (19, 20).

In emerging adulthood, optimal ER relates to college transitions through social competence, self-control, and academic achievement (21). ER is an excellent treatment target in this population because of the widespread effects of dysregulation, especially when emotional suppression can have negative influences on social functioning (22, 23). Focusing on this underlying transdiagnostic mechanism can help prevent multiple negative outcomes. Though existing interventions are efficacious (e.g., Emotion Regulation Therapy, Dialectical Behavior Therapy, Mindfulness-Based Stress Reduction), they often require significant clinician or instructor training and sustained participant engagement. A community-based prevention program targeting ER may achieve meaningful changes in ER that promote participants' wellbeing while reducing time and cost barriers for both instructors/clinicians and participants.

The Gross (24) model of ER provides a foundation for understanding the pathway of emotion generation and regulation. Gross (24) describes a path to an emotional response in which a situation calls forth attention, leading to appraisal and an eventual response. Along this pathway, there are five regulatory strategies: situation selection (e.g., choosing to engage or avoid), situation modification (e.g., changing a situation), attentional deployment (e.g., shifting attentional resources), cognitive change (e.g., modifying thoughts), and response modulation (e.g., adapting behaviors) (25). These may be implemented at any stage of emotion expression to change an emotional state. Houck et al. (26) extrapolated this model to develop a 12-week (24 session) adolescent ER program that successfully reduced risky sexual behaviors. These promising results support the applicability relevance of the Gross (24) model for emerging adults (26), potentially more than adolescents, as cognition and cognitive control become more critical for regulating emotions in emerging adulthood (27).

Mental health is often divided into mental illness and wellness, though these are better conceptualized as ends of a spectrum than dichotomous categories (3). Considerable research on prevention and intervention promotes wellness in emerging adulthood above the absence of disease (28, 29). As a system, colleges have invested in campus counseling and academic centers to provide individual treatment (30–32). Current investments in university counseling centers and mental health prevention programs remain insufficient as more than 25% of college students in need do not have adequate access mental health services (15).

Developing prevention programs requires careful consideration of program delivery, engagement, and reception by the intended audience (33). Online programs require considerations of scalability and accessibility in addition to program effectiveness, as dropout and other barriers such as self-reliance, awareness of symptoms, inadequate knowledge of psychological resources, and stigma may reduce impact (34–36). The RE-AIM framework was developed as a public health guideline for creating effective treatments that can be scaled out and implemented on a large scale (37, 38). Reach (e.g., recruit large/representative portion of target populations), Efficacy (e.g., intervention impact on behavioral target), Adoption (e.g., which settings and staff initiate/participate in program delivery), Implementation (e.g., fidelity to delivery and costs), and Maintenance (e.g., extending treatments broadly, long term effects) are described as the critical pieces of RE-AIM for developing interventions that can be adopted on a larger scale (37, 39).

Ensuring appropriate Implementation, Reach, and Efficacy requires actively engaging the target population and testing the program for intended and unintended consequences, eliciting feedback at multiple levels. Brief ER programs have yet to be developed for emerging adults; therefore, this study takes careful steps to produce an effective and sustainable practical program. After initial content development, scalability was enhanced by acquiring expert and target population feedback. Incorporating feedback from the onset helps ensure that the program will be adopted and well-received. This framework is consistent with participatory action research, integrating reflection and participant input while seeking to reduce inequity and appropriately engage target populations (40).

Intervention efforts are shifting to brief and electronic formats to combat costs and address growing needs for treatment (41–43). Electronic formats for treatment are increasingly popular for youth raised in a digital world (44). Electronic interventions can often overcome typical barriers to care (e.g., accessibility, cost, stigma) at the cost of a potential loss of connection and accountability that mental health professionals provide (45–47). Furthermore, online treatments simplify the incorporation of measurement feedback systems [MFS; (48)]. In a community-based program, incorporating a MFS for continuous progress monitoring can help rapidly identify participant experience problems and diagnose program component reception in the moment. Brief online interventions have proven efficacious in college students for alcohol addiction (49–53), depression (54–56), and anxiety (57–60). Research comparing internet-based and face-to-face clinical interventions has shown equivalent overall effects, though acceptance of online interventions varies by clinician (41, 61–63).

The overall goal of this project was to systematically develop an online brief emotion regulation training (BERT) program designed to improve ER for emerging adults. Since BERT is a novel ER intervention delivered entirely online, this study aims to examine BERT's initial feasibility in preparation for a future randomized controlled trial. Additionally, it aimed to conduct preliminary tests of its Implementation, Reach and Efficacy. This study focused on the creation of BERT utilizing the brainwriting premortem method to refine program content to prepare for initial testing. The program was then tested in a college sample to evaluate implementation in delivering program content, reach to the recruited population, and preliminary efficacy through improved ER. This study was approved by the university institutional review board (IRB#20-018).

### Intervention Development

To develop program content and design, the first author coordinated a team of data scientists, interaction and user experience designers, psychologists, and public health experts. The design team was mindful of guidelines for online design to meet standards of the 1990 Americans with Disabilities Act to ensure broad accessibility. The following sections outline the design of the BERT program and its four primary components: Emotion Regulation Orientation (ERO), Emotion Regulation Training (ERT), Self-Monitoring (SM), and Ecological Momentary Assessment (EMA) based on the Gross model (24).

#### Emotion Regulation Orientation

Participants completed an interactive 30-min online ERO at baseline describing the Gross (24) model of ER (see Supplementary Figure 1) and mental wellness resources (e.g., academic counseling, psychological support).

#### Emotion Regulation Training

Following the ERO, participants began the 5-week ERT. Each week, the ERT used short activities to break down a single ER strategy typically taught in one therapy session. Monday provided psychoeducation, Tuesday applied the strategy to daily life, Wednesday aimed to increase knowledge of the strategy, and Thursday's activity enhanced flexible implementation of these strategies (see Table 1 for ERT exercises).

**Table 1:** Daily emotion regulation training (ERT) program content is outlined below.

**Week**	**Gross (24) Model (*Content area*)**	**Psychoeducation** ***Foundational information provided in the following content areas***	**Gross (24) Model Activity *The ER model is provided step by step over the course of 5 weeks, with each week adding different skills in the emotion regulation process. Participants use examples from their current experiences***.	**Practice 1** ***This is represented in an interactive exercise to practice using the ER model or specific skill*.**	**Practice 2 *This reinforces or summarizes skills learned earlier in the week or continues practice using the ER model or specific skill*.**
1	Situation selection (*Stressor identification and values*)	Stress, decision-making, and values	Identify situation, stressors, physical symptoms, associated values	Identify physical stress in the body, related emotions	Decode provided situation, rate stressors, pick least stressful situation
2	Situation modification (*Problem solving*)	Stressful situations, problem solving, changing situations	Identify situation, stressors, modifications to situation and stressors	Identify possible modifications to short scenarios	Problem solving puzzle: create your own adventure/ change the story
3	Attentional deployment (*mindfulness*)	Mindfulness, shifting attention	Identify situation, stressors, and ways to shift attention from stressors	Stressful situation presented, identify components without stress	“Take 5” grounding exercise (identifying senses)
4	Cognitive change (*CBT skills*)	Cognitive triangle, functional analysis	Identify situation, stressors, antecedents, behaviors, and consequences	Identify thoughts, behaviors, emotions for presented stressful situation, and place on cognitive triangle	Pick out thoughts, behaviors, emotions from provided scenario to interactive cognitive triangle, identify and change antecedents
5	Response modulation (*Healthy regulation* and *coping skills*)	Responding to unmanageable situations	Identify situation, stressors, thoughts, behaviors, emotions, coping strategies	Match list of coping skills to emotional states	Name favorite coping skills, practice present-moment implementation

*ERT is specified for each day*.

#### Self-Monitoring

Participants filled out weekly SM surveys at the start of each ERT week measuring emotional distress, substance use, vitality, exercise, sleep, progress toward goals, and presence/absence of major stressors that could affect functioning. Critical scores triggered delivery of help-seeing resources.

#### Ecological Momentary Assessment

Additionally, EMAs had participants label their emotions and rate their stress in the moment after ERT exercises, though they could be accessed at any time to track their daily mood. The EMA survey was open daily from 8 a.m. until 10 p.m.

#### Intervention Dosage

After initial ERO, participants were asked to complete ~50 min of content in total per week (ERT), in addition to ~25 min of measurement per week. Altogether, the full treatment dose was ~6.75 h of intervention.

### Preliminary Study

#### Premortem Focus Groups

Following recommendations for early engagement of program stakeholders (64) pre-implementation focus groups were conducted for *a priori* identification of program failures (65). Brainwriting premortem methods are more advantageous for evaluating a plan's success than pro/con generation, as they specifically address how a program is designed to fail (66). The brainwriting premortem approach provides an opportunity for psychological safety where data are collected in written form. To accomplish virtual brainwriting premortem within the COVID-19 pandemic, undergraduate focus groups were extended to a 1.5-h online session via videoconferencing software and anonymous contributions to a shared Google document. Clinicians attended 45-min individual interviews *via* videoconferencing software with notes taken by a research assistant during the interview.

Participants included 12 undergraduate students from three universities across four focus groups that took place between June and August 2020. The sample was predominantly female [*n* = 10; male (*n* = 1), trans male (*n* = 1)], heterosexual [*n* = 8; bisexual (*n* = 2)], queer (*n* = 1), lesbian (*n* = 1)], and white [*n* = 7; Asian (*n* = 2), American Indian/Alaskan Native/Latinx (*n* = 1), multiracial (*n* = 2)]. All were full-time students and were in their 2nd (*n* = 2), 3rd (*n* = 2), 4th (*n* = 7), or 5+ year (*n* = 1), with 2 transfer students. In addition, four white women clinicians from early- to mid-career were interviewed of whom two had doctorates in clinical psychology (PhD) and two were licensed clinical social workers (LCSW). All had experience working with emerging adults. Undergraduates and clinicians were all compensated with $10 Amazon gift cards for their participation.

The first author led a team of five trained undergraduate coders to identify themes regarding potential barriers. For each theme, changes to the program were identified in accordance with participant feedback, or a pragmatic or theory-based reason was noted for changes that could not be made (see Table 2). Themes included various skills (e.g., acceptance, coping), language (e.g., increase clarity, inclusivity), timing (e.g., longer or shorter), and external supports (e.g., discussion boards) among others. Though all specific program elements could not be presented in the focus groups and interviews due to time limitations, some themes (29% of undergraduate themes, 26% of clinician themes) were already included in the initial program design. All other themes that could be immediately addressed were incorporated in BERT before initiating the pilot study described below. On a follow-up questionnaire, participants rated their likeliness of completing program content as high (*M* = 4.00, *SD* = 1.13, range = 2–5 on a 5-point scale), and likelihood of recommending BERT to others even higher (*M* = 4.67, *SD* = 0.65, range = 3–5 on a 5-point scale).

**Table 2:** Number of changes across undergraduate focus groups and clinician interviews.

	**Students**			**Clinicians**		
	**Change**	**Concern**	**Keep**	**Change**	**Concern**	**Keep**
Now	10	9	0	8	2	1
Later	9	1	0	18	5	0
None	14	19	0	3	5	1
Embedded	22	18	0	13	6	0
Survey	11	25	0	5	7	0
Compliment	0	0	13	0	0	19
Total	66	72	13	47	25	21

*Responses are coded as (a) address in survey at the end of BERT (1; survey); (b) change now (now); (c) change in later program iterations (later); (d) already in the program, and where (embedded); or (e) don't change, and why (none)*.

## Method

### Participants

A priori power analyses were conducted with G Power v3.1.9.2 (67) determining that a sample size of 36 yields power of 0.90 to complete planned repeated measures ANOVAs. As such, a new sample of undergraduate students (*N* = 42) aged 18–23 years (*M* = 18.88; *SD* = 1.25) was recruited in September 2020 to continue evaluating Implementation, Reach, and Efficacy. Participants were predominantly female (*n* = 36), non-Hispanic white (*n* = 31), heterosexual (*n* = 38), and first-year students (*n* = 22; see Table 3). Participants were recruited through Introductory Psychology classes using an internal extra credit system, academic classes, diversity offices, student centers, women's centers, and wellness centers to encourage recruitment of participants from diverse backgrounds (e.g., underrepresented racial/ethnic groups, sexual minorities, low income) to better evaluate Reach. Students enrolled in undergraduate coursework who did not receive a ceiling score on the DERS were eligible for participation, which took place entirely online. Participants provided informed consent electronically and were compensated with $10 Amazon gift cards at the end of the study. Recruitment completed in October 2020 when participants who enrolled in the study were engaged in the structured program content from October to December 2020. Program content was delivered synchronously to participants who all started week 1 of the ERT simultaneously.

**Table 3:** Demographics for initial enrollment compared to the final sample.

**Identity**	**Initial enrollment**	**Final Sample**
**Gender**		
Female	36	27
Male	6	3
**Sexual orientation**		
Heterosexual	38	28
Bisexual	1	0
Gay	1	1
Don't know	1	1
Multiple sexualities	1	0
**Race**		
American Indian/Alaskan native	1	1
Asian	3	3
Black or African American	2	0
White	31	22
Multiracial	4	3
**Ethnicity**		
Hispanic	2	1
Non-Hispanic	40	29
**Years in college**		
1	22	16
2	10	8
3	4	3
4	3	2
5	2	0
6	1	1

### Measures

#### Attitudes

Participants filled out a questionnaire on their attitudes about BERT during the final survey. These questions were free-response and Likert-scale items which are presented in Supplementary Table 1.

#### Difficulties With Emotion Regulation Scale

This 36-item self-report questionnaire (68) is designed to measure ER in six subscales: non-acceptance of emotional responses (NONACCEPT; α = 0.87–0.91), difficulty engaging in goal-directed behavior (GOALS; α = 0.87–0.89), impulse control difficulties (IMPULSE; α = 0.75–0.82), lack of emotional awareness (AWARE; α = 0.86–0.92), limited access to ER strategies (STRAT; α = 0.87–0.91), and lack of emotional clarity (CLARITY; α = 0.80–0.82), as well as the total score (α = 0.93–0.94), all of which showed good to excellent internal consistency across time points (see Table 4).

**Table 4:** Pilot study descriptive statistics and scale reliabilities for the full sample prior to data analysis or manipulation.

**Survey**		**Initial**				**Midpoint**				**Final**			
**Scale**	**Subscale**	**Mean (SD)**	**Skewness (SE)**	**Kurtosis (SE)**	**α**	**Mean (SD)**	**Skewness (SE)**	**Kurtosis (SE)**	**α**	**Mean (SD)**	**Skewness (SE)**	**Kurtosis (SE)**	**α**
BASE-6	Total	20.26 (9.11)	−0.11 (0.37)	−1.34 (0.72)	0.92	19.10 (8.31)	−0.11 (0.37)	−1.01 (0.83)	0.91	14.64 (6.19)	0.70 (0.44)	−0.14 (0.86)	0.85
DASS-21	Depression	6.57 (6.14)	1.67 (0.36)	3.83 (0.71)	0.85	8.07 (8.53)	0.88 (0.46)	−0.63 (0.89)	0.89	5.71 (6.22)	1.49 (0.44)	1.98 (0.86)	0.82
	Anxiety	4.48 (4.71)	1.67 (0.37)	3.59 (0.72)	0.43	7.08 (6.10)	0.51 (0.46)	−0.78 (0.89)	0.66	4.21 (4.22)	0.79 (0.44)	−0.54 (0.86)	0.53
	Stress	8.73 (5.87)	1.81 (0.37)	5.00 (0.73)	0.51	13.31 (7.67)	0.20 (0.46)	−0.92 (0.89)	0.74	10.43 (8.00)	1.00 (0.44)	1.06 (0.86)	0.85
DERS	Strategies	17.83 (6.74)	0.80 (0.37)	−0.14 (0.72)	0.88	17.46 (7.44)	0.54 (0.46)	−0.95 (0.89)	0.91	15.43 (5.78)	0.83 (0.44)	−0.51 (0.86)	0.86
	Non-acceptance	15.05 (6.62)	0.78 (0.37)	0.02 (0.72)	0.93	14.23 (6.41)	0.75 (0.46)	−0.24 (0.89)	0.92	13.29 (6.58)	1.12 (0.44)	0.37 (0.86)	0.95
	Goals	15.64 (5.47)	−0.27 (0.37)	−1.09 (0.72)	0.89	15.46 (4.75)	−0.26 (0.46)	−0.12 (0.89)	0.87	13.18 (4.14)	−0.40 (0.44)	−0.50 (0.86)	0.89
	Impulse	11.79 (4.64)	0.83 (0.37)	−0.12 (0.72)	0.86	11.00 (3.26)	0.38 (0.46)	0.11 (0.89)	0.75	10.04 (3.55)	0.72 (0.44)	−0.51 (0.86)	0.83
	Awareness	16.42 (5.57)	−0.05 (0.37)	−0.54 (0.72)	0.88	16.43 (4.96)	0.39 (0.46)	0.46 (0.89)	0.86	16.25 (5.53)	0.03 (0.44)	−0.34 (0.86)	0.92
	Clarity	12.67 (4.31)	0.58 (0.37)	−0.24 (0.72)	0.84	12.27 (3.61)	0.52 (0.46)	0.10 (0.89)	0.82	11.46 (3.23)	0.45 (0.44)	0.22 (0.86)	0.81
	Total	87.14 (24.87)	0.39 (0.37)	−0.09 (0.72)	0.95	84.69 (20.39)	−0.20 (0.46)	−0.20 (0.89)	0.93	77.57 (19.67)	−0.12 (0.44)	−0.55 (0.86)	0.93
ERQ	Cognitive reappraisal	27.67 (6.47)	−0.55 (0.37)	−0.37 (0.72)	0.85	27.67 (6.47)	−0.51 (0.46)	0.68 (0.89)	0.70	30.32 (5.71)	−0.52 (0.44)	0.79 (0.86)	0.87
	Expression suppression	16.62 (5.24)	−0.46 (0.37)	0.02 (0.72)	0.81	15.92 (5.05)	−0.54 (0.46)	0.28 (0.89)	0.76	16.25 (5.05)	−0.14 (0.44)	0.14 (0.86)	0.76
MDES	Positive affect total	24.90 (9.69)	0.16 (0.37)	−0.52 (0.72)	0.92					25.12 (10.74)	0.13 (0.44)	−1.00 (0.86)	0.94
	Negative affect total	12.19 (7.78)	0.49 (0.37)	−0.82 (0.72)	0.91					5.54 (5.56)	1.21 (0.44)	0.71 (0.86)	0.84

*Data is presented for participants who completed the initial (n = 41–42), midpoint (n = 26–30) or final (n = 28) survey*.

#### Emotion Regulation Questionnaire

This 10-item self-report questionnaire (69) has two subscales: cognitive reappraisal (CR; α = 0.70–0.89) and expressive suppression (ES; α = 0.76–0.82; Table 4) which showed good internal consistency across time points.

#### Brief Adjustment Scale

This 6-item self-report questionnaire (70) measures overall psychological functioning. It showed good to excellent internal consistency across time points (α = 0.84–0.91; see Table 4).

#### Modified Differential Emotion Scale

This 20-item self-report measure (71) assesses positive and negative affective experiences. Good to excellent internal consistency across time points was shown for the positive (α = 0.92–0.99) and negative affect (α = 0.85–0.90) scales (see Table 4).

#### COVID-19 Scales

This series of measures (72) was adapted from recommended CDC scales to measure impacts due to the COVID-19 pandemic. Three scales were utilized in this study: Perceived Coronavirus Threat Questionnaire (PCTQ; α = 0.82), Coronavirus Impacts Questionnaire (CIQ; α = 0.70), and the Coronavirus Experiences Questionnaire (CEQ; α = 0.69). All exhibited acceptable to good internal consistency. The CIQ is divided into three subscales: financial (α = 0.54), resource (α = 0.90), and psychological (α = 0.83). The CEQ is divided into 3 subscales: personal diagnoses/symptoms (α = 0.55), proximity to others (α = 0.86), and news (α = 0.60; see Table 4).

### Procedure

Participants completed initial (prior to ERO), midpoint (week 3), and final (following week 5) Qualtrics surveys to evaluate impacts of BERT on ER and psychological distress. All participants received content for ERT Week 1 on the same date, to ensure all participants were completing the program on the same timeline. For self-monitoring, participants completed a brief questionnaire including the BASE-6 and vitality subscale of the Thriving Scale (73) at the start of each week. Automatic feedback was provided with interpretation, graphing scale scores and self-monitoring reports of sleep and substance use. Graphed individual feedback could be viewed by participants on a password protected, Google Apps webpage which ingested individual participant data from Qualtrics (see Supplementary Figure 2). Every day participants received the mDES as an EMA to capture daily mood. This measurement was designed to enhance emotion identification and emotional granularity. Alongside this measurement, participants also engaged in the 5-week ERT curriculum. Participation was recorded through their responses to the Qualtrics survey, serving as a manipulation check. In other words, BERT engagement was tracked by participants' responses to the daily program content.

## Results

### Data Cleaning

**Figure 1:**
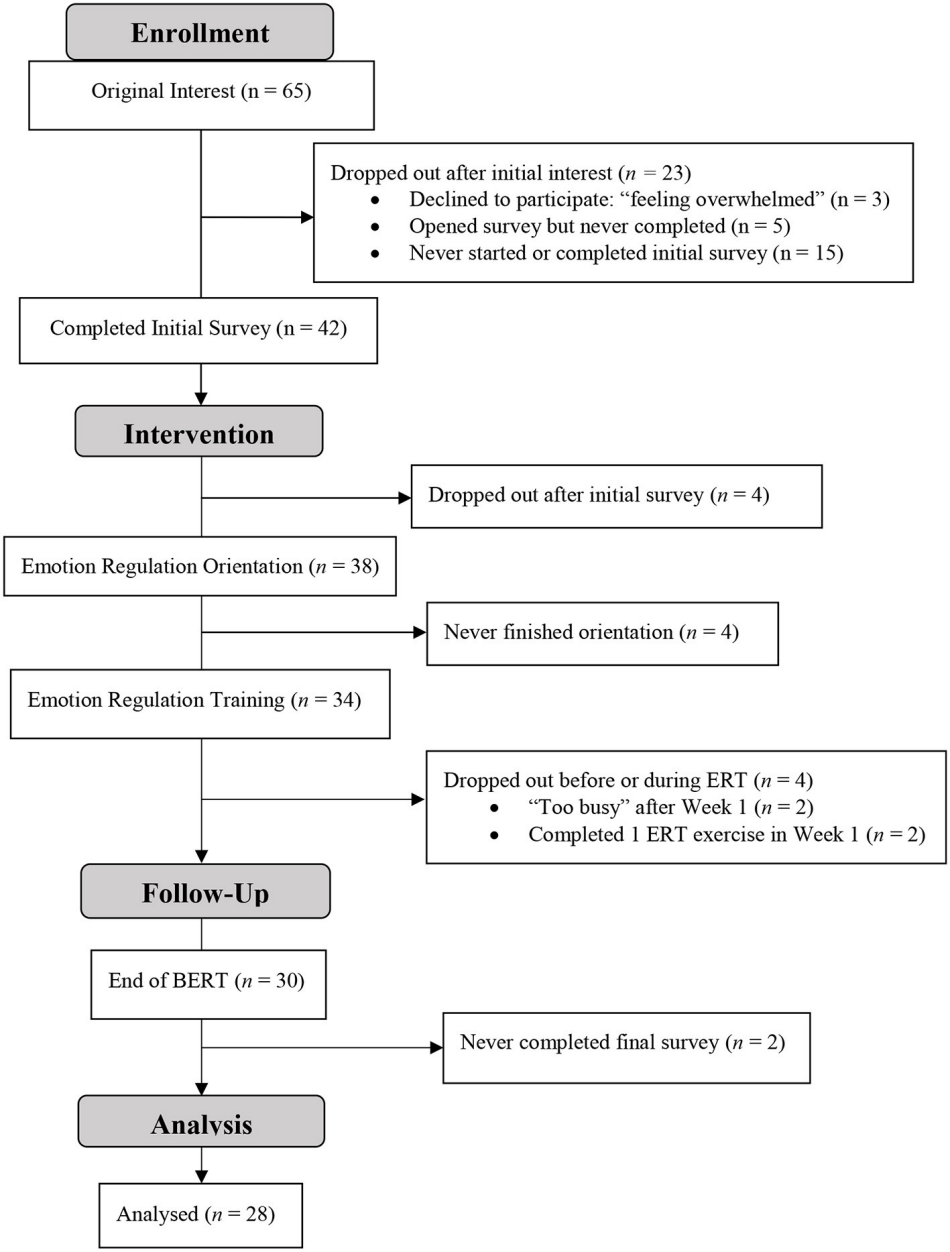
Consort diagram to examine BERT completion rates and attrition.

Data was screened for univariate and multivariate outliers; none were identified. Three participants did not complete the midpoint survey. Little's MCAR Test was performed to determine if values were missing at random [$\chi_{\left(147,\text{N}=42\right)}^{2}$ = 106.80; *p* = 0.995]. Missing midpoint values were replaced by carrying forward initial survey values, providing a conservative estimate assuming no change. Two participants who completed BERT did not complete the final survey, with an additional participant missing parts of the survey. Little's MCAR Test was performed [$\chi_{\left(129,\text{N}=30\right)}^{2}$ = 104.57; *p* = 0.944] and missing final values for these 3 participants were replaced by carrying forward midpoint survey values, or initial survey values if they did not complete the midpoint survey. The overall attrition rate was 11.8%. Participants initiated BERT if they completed the ERO prior to the first week of the ERT (34 of the 42 participants) and completed BERT if they completed program content through Week 5 (*n* = 30), though 2 completers did not complete the final survey (see CONSORT diagram, Figure 1).

#### Cost

BERT development took a substantial amount of time and had associated costs. Approximately 250 person hours were required for programming and graphic design, in addition to content development time. There were insufficient funds to build this program as an app-based software. Downstream costs associated with the Google Apps Script (GAS) platform included collaborative efforts between the grant team and five undergraduate students to ensure the program was delivered with fidelity. Approximately 1–2 h per day were required by team members to oversee the data collection process, send program content, and manage email reminders.

#### Enrollment

Females were represented in the study at a significantly higher rate (86%) than in the university [43%; $\chi_{\left(1,\text{N}=42\right)}^{2}$ = 14.13; *p* < 0.00; see Table 3]. The ethnoracial distribution of this sample did not significantly differ from the university population. Because the university only reports ethnicity separately from race, results were extrapolated from two calculations: (1) assuming that no White students enrolled in the university during the semester the study took place were Hispanic/Latinx, and (2) assuming that all Hispanic/Latinx students enrolled in the university during the study took place were White. The true value for the population will fall somewhere between these two values. In both cases, the actual numbers of ethnoracial minority students (ERM; *n* = 13) and non-Hispanic White students (NHW; *n* = 29) were not significantly different than expected based on university demographics ([1] ERM = 13.26, NHW = 28.74; $\chi_{\left(1,\text{N}=42\right)}^{2}$ = 0.007; *p* = 0.931; [2] ERM = 16.8, NHW = 25.2; $\chi_{\left(1,\text{N}=42\right)}^{2}$ = 1.433; *p* = 0.231). Thus, the success of Reach was mixed, with representation adequate by race/ethnicity, but not ideal by gender.

#### Engagement

Chi-square analyses were conducted to examine whether there were demographic differences between participants who completed the initial survey and continued on to BERT compared with those who did not initiate BERT. Completion rates for each part of the program are shown in Table 5. Though 38 participants started the ERO, 34 completed it, with 32 beginning the ERT. There were no significant differences based on minority status [$\chi_{\left(1,\text{N}=42\right)}^{2}$ = 35; *p* = 0.55], gender [$\chi_{\left(1,\text{N}=42\right)}^{2}$ = 1.19; *p* = 0.28], or first year status [$\chi_{\left(1,\text{N}=42\right)}^{2}$ = 0.02; *p* = 0.90]. These results support the Reach of BERT. To explore the data, separate logistic regressions were conducted to examine whether there were study variable differences between participants who completed the initial survey and continued to BERT compared with those who did not initiate BERT. There were no significant differences based on age [$\chi_{\left(1,\text{N}=42\right)}^{2}$ = 0.68; *p* = 0.17], BASE-6 [$\chi_{\left(1,\text{N}=42\right)}^{2}$ = 0.13; *p* = 0.72], DERS subscales [$\chi_{\left(6,\text{N}=42\right)}^{2}$ = 2.90; *p* = 0.82], or ERQ subscales [$\chi_{\left(2,\text{N}=42\right)}^{2}$ = 0.58; *p* = 0.75], mDES scales [$\chi_{\left(4,\text{N}=42\right)}^{2}$ = 3.25; *p* = 0.52; see Table 5].

**Table 5:** Number of completed surveys or program content for the ecological momentary assessment (EMA), emotion regulation training (ERT), and self-monitoring (SM).

	**%**	***n***
**EMA completions**		
38	108.6	1
37	105.7	1
36	102.9	1
35	100	3
34	97.1	3
33	94.3	2
32	91.4	4
31	88.6	1
30	85.7	3
28	80	1
27	77.1	4
26	74.3	1
22	62.9	1
19	54.3	1
17	48.6	1
10	28.6	1
9	25.7	1
8	22.9	1
2	5.7	1
0	0	2
	***N***	**34**
**ERT completions**		
18	100	20
17	94.4	4
16	88.9	2
14	77.8	1
13	72.2	1
12	66.7	1
9	50	1
7	38.9	1
3	16.7	1
1	5.6	2
	***N***	**34**
**SM completions**		
5	100	9
4	80	16
3	60	4
2	40	1
1	20	1
0	0	3
	***N***	**34**

Technical errors for collecting compliance, had ERT completion rates based on 18 exercises (*M* = 16.57; *SD* = 2.81). Nineteen participants completed 100% of the ERT content, with 26 (78.1%) completing more than 75% of the ERT exercises. Participants may not have had equal opportunities to complete all 5 SM surveys due to a technical error in Week 4, so the 75% criterion was set at 3 SM completions with 85.3% of participants completing more than 75% of the SM opportunities. There were 35 EMA opportunities (daily for 5-weeks), and the average number of EMAs completed was 28 (80% completion rate), and 70.6% (*n* = 20) of participants exceeded a 75% completion rate. Three participants completed the EMA more than 35 times. Thus, engagement data support Implementation of BERT.

#### Attitudes

Descriptive statistics for participants' attitudes about BERT reported on the final survey are presented in Supplementary Table 1. Overall, participants' responses suggest favorable attitudes, as all positively worded items had mean scores above the mid-points on their respective scales and all negatively worded items had mean scores below the mid-points on their scales. Notably, all components of BERT were reported as helpful (*Ms* = 5.29–5.79 on a 7-point scale). There were varied responses to the ease of remembering the program concepts (*M* = 4.61; *SD* = 1.52 on a 7-point scale) and application to daily life (*M* = 4.89; *SD* =.96 on a 7-point scale). Participants reported that it was not difficult to keep up with program demands (*M* = 3.36; *SD* = 1.34 on a 7-point scale), that they were engaged (*M* = 6.11; *SD* = 0.79 on a 7-point scale), and the wording was easy to understand (*M* = 6.04; *SD* =0.69 on a 7-point scale). Participants reported they would be somewhat likely to recommend BERT to someone else (*M* = 3.86; *SD* = 0.59 on a 5-point scale). All of this supports the Implementation of BERT.

Independent samples *t*-tests were conducted to examine whether there were race/ethnicity (minority vs. White) differences in participants' favorable attitudes toward BERT on the follow-up survey. There were no significant differences in attitudes about helpfulness (*p*s = 0.07–0.93). There were no differences in difficulty remembering program concepts [*t*_(25)_ = −0.93; *p* = 0.16], applicability to daily life [*t*_(25)_ = −1.27; *p* = 0.22], keeping up with program demands [*t*_(25)_ = 0.80; *p* = 0.71], engagement [*t*_(25)_ = −0.46; *p* = 0.60], understanding wording [*t*_(25)_ = −0.77; *p* = 0.15], or recommending the program to others [*t*_(25)_ = 0.13; *p* = 0.97]. A similar *t*-test could not be conducted to test for gender differences because too few men completed BERT. The absence of differential attitudes based on ethnic/racial identity suggests no barriers to Reach.

#### Emotion Regulation

As the Bonferroni correction was utilized, the corrected alpha for the DERS was 0.007 and for the ERQ was 0.025. Repeated measures ANOVA showed overall difficulties with emotion regulation (DERS total score) significantly decreased from baseline to follow-up [*F*_(2,58)_ = 9.07; *p* < 0.000; partial η η^2^ = 0.24; power = 0.97], consistent with hypotheses. Based on the Bonferroni-corrected alpha, no significant change was noted for the subscales: limited access to emotion regulation strategies [*F*_(2,58)_ = 5.07; *p* = 0.009; partial η^2^ = 0.15; power = 0.80]; non-acceptance of emotional responses [*SD* = 4.15; *F*_(2,58)_ = 5.11; *p* = 0.009; partial η^2^ = 0.13; power = 0.78]; difficulty engaging in goal-directed behavior [*F*_(2,58)_ = 5.64; *p* = 0.009; partial η^2^ = 0.29; power = 0.82]; impulse control difficulties [*F*_(2,58)_ = 4.52; *p* = 0.015; partial η^2^ = 0.14; power = 0.75]; lack of emotional awareness (*F*_(2,58)_ = 0.14; *p* = 0.870; partial η^2^ = 0.01; power = 0.07]; lack of emotional clarity [*F*_(2,58)_ = 3.82; *p* = 0.028; partial η^2^ = 0.12; power = 0.67].

On the ERQ, cognitive reappraisal skills significantly improved from baseline to follow-up [*F*_(2,58)_ = 7.73; *p* < 0.001; partial η^2^ = 0.21; power = 0.94], consistent with hypotheses, but there was not power to detect a similar statistically significant reduction in emotion suppression over time [*F*_(2,58)_ = 2.49; *p* = 0.092; partial η^2^ = 0.08; power = 0.48].

#### Psychological Distress

Repeated measures ANOVA showed that psychological distress assessed on the BASE-6 significantly decreased from baseline to follow-up [*F*_(2,58)_ = 8.42; *p* < 0.001; partial η^2^ = 0.23; power = 0.96]. Additionally, the predicted cutoff score for this measure is 19 (70), suggesting that the decrease from 20.50 to 14.97 also is a clinically meaningful decrease in scores.

#### mDES

As the Bonferroni correction was utilized, the alpha for the mDES was 0.025. Changes in mDES total positive and negative affect scores were examined between baseline and follow-up with a repeated-measures ANOVA to determine broader changes in affectivity following BERT engagement. There was no change from baseline to follow-up in positive affect [*F*_(1,29)_ = 0.02; *p* = 0.882; partial η^2^ = 0.00; power = 0.05]. There was a significant decrease in negative affect over time [*F*_(1,29)_ = 13.07; *p* = 0.001; partial η^2^ = 0.31; power = 0.94].

#### COVID-19

The COVID-19 pandemic caused a range of interference in students' lives as evidenced by scores on the PCTQ, CIQ, and CEQ (see Table 6). When compared to outcome variables, the CIQ was positively correlated with DERS, BASE-6, and mDES and the CEQ was positively correlated with the DERS (see Table 6).

**Table 6:** Descriptive statistics on the short CDC COVID-19 scales: PCTQ, Perceived Coronavirus Threat Questionnaire; CIQ, Coronavirus Impacts Questionnaire; CEQ, Coronavirus Experiences Questionnaire.

**Scale**	**Subscale**	**Range**	**Mean (SD)**	**Skewness (SE)**	**Kurtosis (SE)**	
PCTQ	Total	3–17	9.78 (4.35)	−0.30 (0.45)	−1.07 (0.87)	0.82
CIQ	Total	6–33	15.67 (6.11)	0.62 (0.45)	0.99 (0.87)	0.70
	Financial	2–14	5.04 (2.82)	1.34 (0.45)	2.61 (0.87)	0.54
	Resources	2–10	4.22 (2.65)	1.09 (0.45)	0.01 (0.87)	0.90
	Psychological	2–12	6.41 (3.18)	0.27 (0.45)	−1.23 (0.87)	0.83
CEQ	Total	7–47	20.37 (12.01)	0.99 (0.45)	0.11 (0.87)	0.69
	Diagnoses/symptoms	3–20	7.81 (6.34)	1.04 (0.45)	−0.55 (0.87)	0.55
	Proximity	2–18	7.48 (6.39)	0.77 (0.45)	−1.04 (0.87)	0.86
	News	2–16	5.07 (3.68)	1.55 (0.45)	2.15 (0.87)	0.60

*Subscales for the CIQ include: Financial Scale, Resource Scale, Psychological Scale. Subscales for the CEQ include: Personal Diagnoses/Symptoms Scale (Diagnoses/Symptoms), Proximity to Others Scale (Proximity), News Scale*.

## Discussion

An online Brief Emotion Regulation Training (BERT) program was developed and evaluated within the RE-AIM framework (37) to ensure this program is both efficacious and scalable. BERT was adapted based on focus groups and clinician feedback prior to pilot testing to determine its Implementation, prospective Reach, and Efficacy. Though there was substantial attrition between soliciting interest and initiating BERT, Implementation was mostly supported, and initial Efficacy was promising. BERT demonstrated Reach regarding race/ethnicity, but not gender, suggesting the importance of future program development exploring the barriers and preferences of undergraduate men for developing emotion regulation skills.

BERT's strength is as a brief and intensive online intervention that does not require the presence of a clinician. Taking a transdiagnostic and preventive lens, BERT focuses on ER as an underlying mechanism impacting a wide range of psychopathology in emerging adulthood. Additionally, BERT is sensitive to a health equity framework (74), attempting to circumvent traditional barriers to care and provide equitable care to vulnerable populations. The brief and intensive nature of this program is designed to be more immersive than traditional interventions (42). Each component was designed to mimic face-to-face evidence-based treatments. BERT was designed to build emotion regulation strategies guided by the Gross (24) process model of ER. Influenced by a cognitive-behavioral perspective, the program incorporates third-wave components (i.e., mindfulness, acceptance). Specifically, the orientation provides a grounding in psychoeducation on ER and mental health, the weekly self-monitoring is a weekly symptom check-in akin to measurement-based care (75), the EMA is intended to enhance emotional clarity, and the ERT is aimed at facilitating daily ER skills practice. While these components were delivered together, future iterations will disentangle these components to understand which ones are most significantly contributing to change.

### Premortem Focus Groups

Incorporating stakeholders to identify changes and concerns early in BERT's development provided invaluable input for developing a sensitive and targeted treatment program. Pragmatically, the brainwriting premortem simplified qualitative data collection, as participants shared and responded to ideas in writing. Many suggested changes were already in the program, but critical observations by participants were incorporated prior to pilot launch, including steps for behavior change, types of coping, windows of completion, sensitive language, and general clarity. Further BERT development will integrate ancillary premortem and initial feedback to enhance the applicability of program content.

### Reach

Promising initial results supported BERT's utility. Recruitment showed initial interest in BERT, though ~1/3 of students screened did not continue to the initial survey, roughly consistent with dropout rates in studies of smartphone apps for depression (76). Most participants who engaged past the first week of ERT were still active in the final week. There was mixed evidence for BERT's Reach. Students with minoritized racial and ethnic identities participated at rates that would be expected given the university distribution, representing a strength in Reach. Ensuring equitable Reach is especially important because these students are likely to face race-related stressors that increase health risks (77). Strengthening emotion regulation skills may ameliorate physiological and psychological mechanisms for the detrimental effects of race-related stressors, thereby promoting health equity (Peterson et al., 2020). The female-dominated program enrollment did not adequately represent the 57% male overall student population. Gender biases may reduce male-identifying individuals' participation in a program explicitly focused on emotion, as there is still stigma around attending to emotional wellbeing in male-identifying individuals (78–80). There were too few male participants to test gender differences in engagement and favorable attitudes. There were no gender differences in attrition and no race/ethnicity differences in engagement, attrition, or favorable attitudes.

### Implementation

Implementation was supported from participants' engagement, low attrition once initiating BERT, and favorable attitudes. However, there were time costs, technological errors, and known technological limitations that posed barriers to implementation and retention. Relying on Qualtrics and GAS instead of mobile apps limited the program design, preventing use of SMS and push notifications that could have simplified content access and reduced implementation team time cost. Personalizing program reminders and having participants choose their notification preferences (i.e., text, push notification, email) would improve accessibility and reduce confusion. Moreover, participants all had to begin the program at the same time, which could have constrained Reach due to delayed initiation or period-specific life circumstances.

Engagement measures including attrition (12%) and mean content completion (92%) compare favorably with app- or smartphone-based interventions for health behaviors and disease management (76, 81–83). BERT completers generally considered the program helpful, reporting positive impacts on emotional and mental wellbeing. The online format ensured that all participants completed measurements within the same timeframe and allowed participants to choose the location and timing of survey completion within administration windows.

### Efficacy

This study sought to examine initial efficacy to determine whether further randomized controlled trials are indicated. Initial Efficacy of BERT was promising as there was improvement in DERS total and ERQ cognitive reappraisal scores. No significant changes were seen in DERS subscale scores which may reflect low power due to the small sample size or broad, rather than targeted, change in ER. Still, BERT shows significant improvements in overall ER and cognitive reappraisal, suggesting that foundational ER skills are increasing even if participants are not recognizing or reporting an increase in specific ER components. Interestingly, the mDES exhibited significant reductions in negative emotion but no significant change in positive emotion; this may have been limited by higher reports of positive emotion at baseline. Decreases in negative affectivity could result from increased regulation or management of negative emotional states. Unfortunately, a virtual platform does not allow for in the moment clinical judgment. Future research could expand measurement to include emotionally salient tasks, changes in risk-taking, and executive functioning tasks to better understand how BERT impacts specific ER components or skills.

Psychological distress also saw significant reductions, a powerful result in the context of a cascading “mental health pandemic” resulting from the isolating effects of social distancing and quarantining during COVID-19 (84). It is interesting that on average baseline scores on the BASE-6 were clinically elevated and returned to below the clinical cutoff after the BERT intervention. This secondary effect highlights BERT's prevention value and further supports focus on ER as a critical transdiagnostic factor underlying psychopathology in emerging adults. At this stage, it is challenging to conclusively determine efficacy since there is no control group. Future studies will focus on testing with a waitlist control to ensure that changes are not due to random effects or time-series variation.

### COVID-19

An unavoidable limitation was conducting this work during the COVID-19 pandemic., which significantly impacted general mental health and wellbeing (85). As individuals were forced to stay indoors and away from others for safety, ER may have been especially critical to cope with distress. Participants who experienced greater adversity from the pandemic had worse ER and psychological outcomes. Interestingly, receiving a diagnosis of COVID-19 or experiencing symptoms of COVID-19 related to a decrease in anxiety. It is possible that after having the illness, there was less anxiety as there was a reduced fear of becoming infected, or individuals may have felt a sense of relief from the potential to re-open networks after acquiring antibodies. Notably, there were no necessary modifications to the proposed BERT for it to be implemented during the pandemic, highlighting the flexibility of online interventions.

### Limitations

Despite strong recruitment efforts, only 42 participants enrolled in BERT, limiting statistical power. The small, somewhat homogenous, sample also raises generalizability concerns about findings. Additionally, since this program is designed as a preventive intervention it is difficult to disentangle potential adverse effects, though none were observed during this study. Occasional technological errors in content delivery may have also impacted observed results, though statistically more likely to have biased effect estimates downward than upward. Since BERT was not yet fully automated, manual email reminders were sent to keep participants on track. Occasionally, difficulties in loading the participant homepage impacted data collection, making it impossible to know if participants were 100% compliant with program content. Replicating testing of BERT with a larger and more diverse sample and with fully automated program delivery are thus important next steps. This stage of program development focused primarily on implementation and feasibility, thus excluding a control group comparison to maximize statistical power. Future work will benefit from a waitlist control design.

### Conclusions and Future Directions

Overall, results are promising for transdiagnostic treatment approaches to prevention like BERT. Approaching treatment development with the end in mind allows for critical examination of shortcomings and growth areas of BERT. While the online format lends itself to better fidelity, technological barriers still impeded implementation. By identifying and addressing dissemination barriers, BERT can be adapted to facilitate widespread program uptake. BERT takes an upstream approach to addressing a key mechanism underlying multiple forms of psychopathology, allowing it to effectively prevent a range of negative outcomes while promoting wellbeing. BERT can be widely disseminated because it is cost-effective, adaptable, and does not require significant clinician burden. BERT has great potential as a cost-effective tool to promote regulation and mitigate distress in emerging adulthood. Accessibility by individuals with disabilities was emphasized throughout the design process. Future development aims to test BERT with more ethnoracial and gender identity diverse samples to ensure an equitable program. When unmanaged, poor ER can manifest in deleterious ways (e.g., impulsive behaviors, outbursts, criminal behaviors, dropout) with a host of negative associated consequences (86). Proactively promoting ER strategies rather than reactively treating observed deficits can reduce individual and societal costs of mitigating the effects of dysregulation by preventing its emergence. BERT may also be modified for adolescent populations with the hopes of improving ER, reducing risk, and fostering resiliency in vulnerable youth. Integrating BERT into school settings may allow for increased accessibility and accountability, with professional support more readily available for youth in need of assistance with emotion identification and skill development. Additionally, BERT may be useful as a tool for individuals in need who are still waiting to receive clinical care. BERT has the potential to serve as a cost-effective prevention method for improving ER in vulnerable populations, by circumventing traditional barriers to care.

## Data Availability Statement

The raw data supporting the conclusions of this article will be made available by the authors, without undue reservation.

## Ethics Statement

The studies involving human participants were reviewed and approved by Virginia Tech Institutional Review Board. The patients/participants provided their written informed consent to participate in this study.

## Author Contributions

AG came up with the intellectual property and BERT intervention as well as the primary manuscript drafts, which was supervised by LC and JD. SH provided consultation regarding implementation science. The data bridge team including AB, NP, MS, and TE assisted in the development of the technological components of BERT and assisted with data collection. All authors assisted with editing drafts of the manuscript. All authors contributed to the article and approved the submitted version.

## Funding

This project was funded by the Southern Regional Education Board Dissertation Award, the Thomas H. Ollendick Ph.D. and Mary Catherine Haley Ollendick Graduate Fellowship in Clinical Child Psychology, and the Institute for Creativity, Arts, and Technology (ICAT) at Virginia Tech SEAD Grant. Open access publishing fees were supported by the Virginia Tech Open Access Subvention Fund.

## Conflict of Interest

The authors declare that the research was conducted in the absence of any commercial or financial relationships that could be construed as a potential conflict of interest.

## Publisher's Note

All claims expressed in this article are solely those of the authors and do not necessarily represent those of their affiliated organizations, or those of the publisher, the editors and the reviewers. Any product that may be evaluated in this article, or claim that may be made by its manufacturer, is not guaranteed or endorsed by the publisher.
