# Supplementary material for: Development and Feasibility of an Online Brief Emotion Regulation Training (BERT) Program for Emerging Adults
Source: Front Public Health. 2022 Jun 10;10:858370. doi: 10.3389/fpubh.2022.858370 (PMC9226550; doi:10.3389/fpubh.2022.858370)
Supplement: Supplementary file 1 [file Data_Sheet_1.docx]

Table S1. *Attitudes about BERT.*

|  | **Statement** | **Min-Max** | **M (SD)** |
| --- | --- | --- | --- |
| Orientation | The orientation provided information that was new to me. | 3 – 7 | 5.46 (0.79) |
|  | The information provided was unhelpful. | 1 – 7 | 3.11 (1.85) |
|  | The orientation took an appropriate amount of time. | 3 – 6 | 5.50 (0.88) |
|  | The weekly training would be acceptable without this orientation. | 2 – 7 | 4.54 (1.26) |
|  | How difficult was it to complete this orientation? | 3 – 7 | 5.46 (1.23) |
|  | Do you feel that the orientation was helpful? | 3 – 7 | 5.29 (1.01) |
|  | How long would this orientation ideally take, provide your answer in minutes: | 5 – 90 | 20.58 (15.58) to 22.31 (16.57) |
| ERT | How difficult was it to complete the daily activities? | 3 – 7 | 5.86 (0.97) |
|  | How often would you like these activities to occur (in days/week)? | 1 – 7 | 3.41 (1.61) |
|  | How long would these activities ideally take, provide your answer in minutes: | 2 – 25 | 8.89 (4.40) to 10.88 (4.81) |
| Week 1 | How helpful did you find this week’s content to be? | 4 – 7 | 5.46 (0.79) |
| Week 2 | How helpful did you find this week’s content to be? | 2 – 7 | 5.43 (1.03) |
| Week 3 | How helpful did you find this week’s content to be? | 4 – 7 | 5.64 (0.87) |
|  | The create your own adventure was helpful. | 2 – 7 | 5.33 (1.30) |
| Week 4 | How helpful did you find this week’s content to be? | 2 – 7 | 5.79 (1.13) |
| Week 5 | How helpful did you find this week’s content to be? | 4 – 7 | 5.61 (0.79) |
| Self-Monitoring | I looked at the graphs on the BERT homepage. | 1 – 7 | 4.89 (2.04) |
|  | Feedback provided from self-monitoring was useful. | 2 – 7 | 5.18 (1.16) |
|  | I discussed the feedback I received from self-monitoring with other people. | 1 – 6 | 2.39 (1.57) |
|  | How difficult was it to complete monitoring weekly? | 2 – 7 | 5.54 (1.29) |
|  | Do you feel that self-monitoring was helpful? | 4 – 7 | 5.64 (0.99) |
|  | How often would you like these measurements to occur (in days)? | 1.5 – 17.5 | 6.56(3.72) |
|  | How long would these activities ideally take, provide your answer in minutes | 1—30 | 10.68 (7.86) to 11.68 (7.96) |
| EMA | How difficult was it to complete these measures daily? | 2 – 7 | 5.75 (1.27) |
|  | Do you feel that daily measurement was helpful? | 1– 7 | 5.23 (1.61) |
|  | How often would you like these measurements to occur (in days/week)? | 1– 14 | 5.82 (2.91) |
|  | How long would this measurement ideally take, provide your answer in minutes | 2 – 25 | 6.96 (5.64) |
| Overall BERT | I feel that BERT was helpful in improving my emotion regulation. | 3 – 7 | 5.68 (0.98) |
|  | I feel that BERT helped me understand and identify my emotions. | 4 – 7 | 5.68 (0.91) |
|  | I feel that BERT helped me better manage my mental health. | 4 – 7 | 5.5 (0.92) |
|  | I feel that BERT helped me better understand my own mental health. | 4 – 7 | 5.82 (0.72) |
|  | I dislike how brief this program is. | 1– 6 | 5.82 (.72) |
|  | I dislike the intensive nature of this program. | 1 – 6 | 3.57 (1.26) |
|  | I feel the examples provided throughout the program were too cliché. | 2 – 6 | 3.93 (1.36) |
|  | I found it easy to remember the concepts taught in this program. | 1 – 7 | 4.61 (1.52) |
|  | I applied the skills learned in this program to my daily life. | 3 – 7 | 4.89 (0.96) |
|  | I paid attention and was honest when completing the program content. | 4 – 7 | 6.11 (0.79) |
|  | I found it difficult to keep up with the demands of this program. | 1 – 6 | 3.36 (1.34) |
|  | The wording of this program was easy to understand. | 4 – 7 | 6.04 (0.69) |
|  | How clear was this model of emotion regulation? | 1 – 5 | 3.93 (.94) |
|  | I sought out support (e.g., friends, family, professionals) because of BERT. | 1 – 6 | 3.00 (1.61) |
|  | Would you want to attend an in-person while taking this program (outside of a global pandemic)? | 1 – 3 | 2.04 (.74) |
|  | Would you participate in an online discussion board while taking this program? | 1 – 3 | 2.07 (0.86) |
|  | How likely are you to recommend BERT to someone else? | 3 – 5 | 3.86 (0.59) |


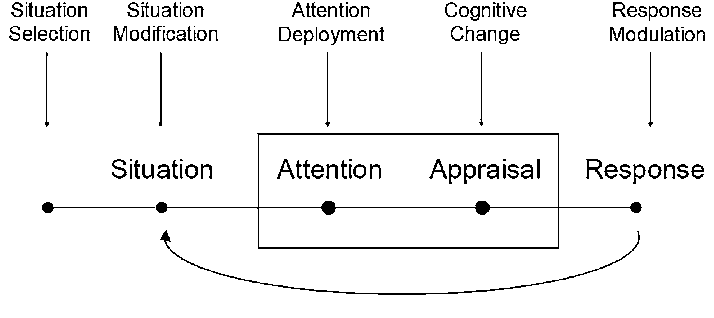
**A**


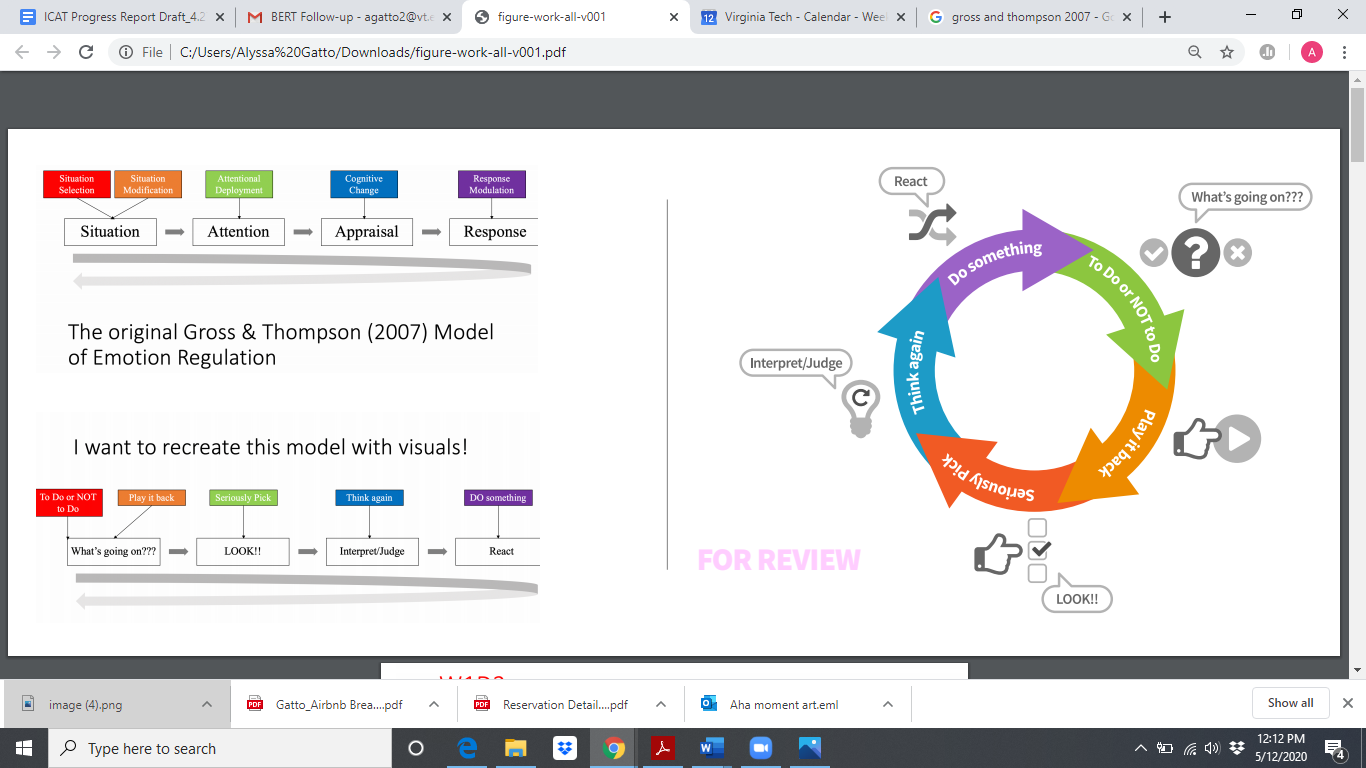


**B**


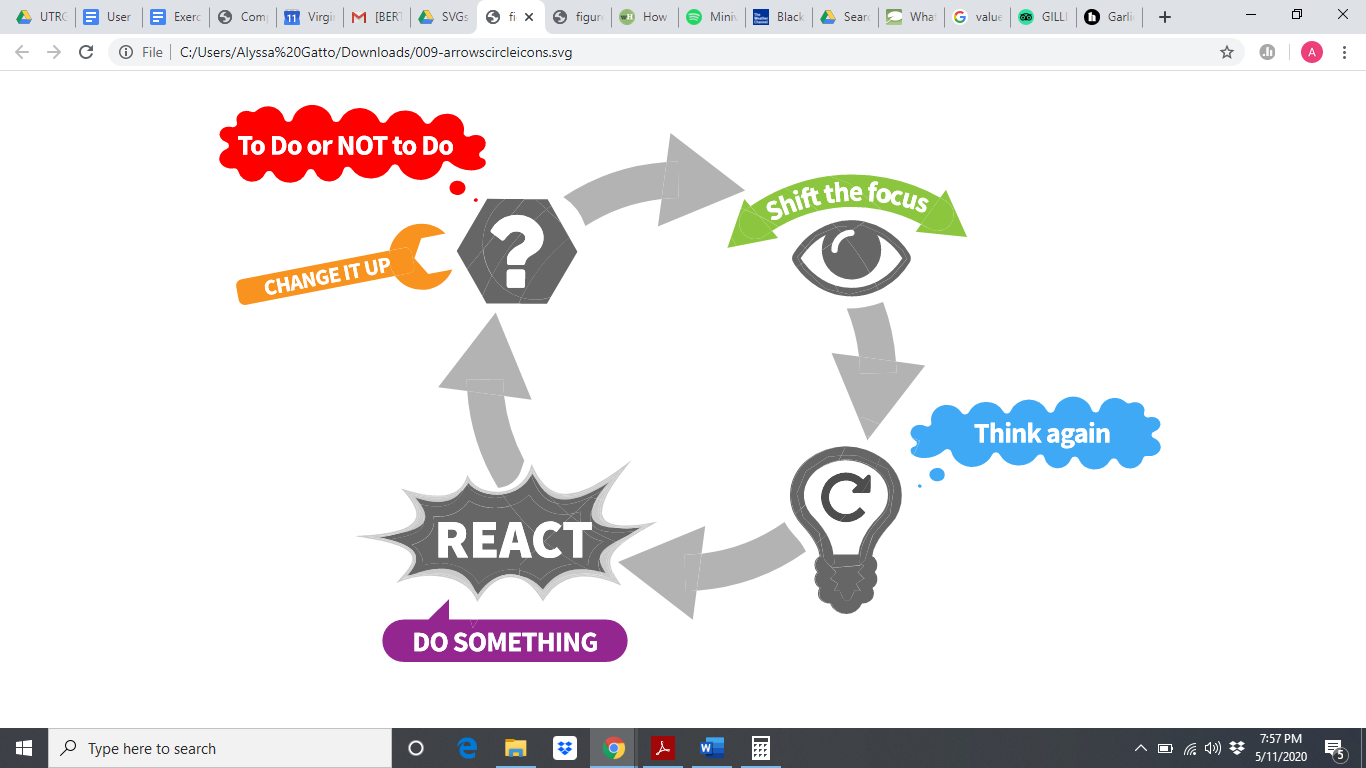


**C**

*Figure S1.* Iterations of the Gross (2014) process model of emotion regulation. (A) Original Gross & Thompson (2007) process model of emotion regulation. (B) Draft and original versions of BERT model of emotion regulation. (C) Current iteration of BERT model of emotion regulation.


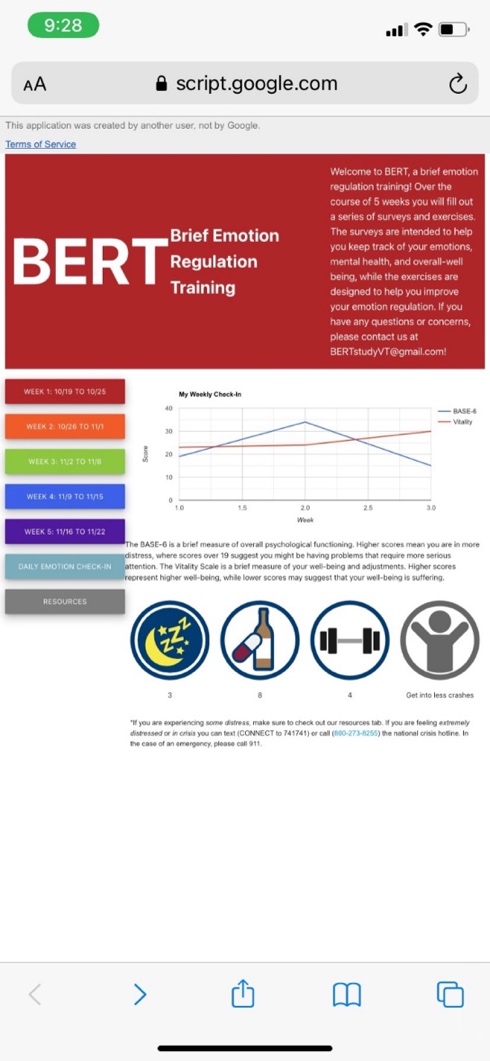


*Figure S2.* Mobile version of the BERT homepage.
